# A Case of Osteo Aneurism (?) Occurring in the Os Calcis; Amputation and Recovery

**Published:** 1853-06

**Authors:** W. Parker

**Affiliations:** Professor of Surgery in the College of Physicians and Surgeons, New York


From the N. Y. Jour. of Medicine.

*A Case of Osteo-Aneurism (?) occurring in the Os Calcis; Amputation and Recovery.* By W. Parker, M. D., Professor of Surgery in the College of Physicians and Surgeons, New York.

In an article on *Pulsatory Tumors of Bone*, in the last number of the *N. Y. Journal of Medicine*, by Dr. C. D. Smith allusion is made to the following case, which occurred in my practice. A similar case occurred at the Emigrant Hospital, under the charge of Dr. Carnochan, and by him was published, with a resume of the subject, in the *N. Y. Medical Gazette* for January 1853. Of these three cases, which comprise, I believe, the experience of the profession in this city, in regard to this form of disease, my own remains unpublished. I wish, therefore, to place it on record, as a contribution to this subject, the history of which has already been creditably given in the papers above referred to.

The patient was an intelligent physician, Dr. Wm. Werden, of Salisbury, who drew up the history of his own case, at my request. The appearances of the morbid specimen have been mentioned by Dr. Smith. At the time of writing his case, the doctor was in the enjoyment of good health; I have more recently learned that he is again on the decline, with well marked symptoms of an advanced stage of pulmonary disease.

Case.—In the summer of 1842, while jumping for recreation, I struck my left heel upon a round stick about an inch in diameter; it gave me severe pain at the time, which, however, lasted but a few minutes, and I thought no more of it. Several days after, upon rising from my bed in the morning, I experienced a sharp pain darting through my heel, which quickly passed off, but recurred again on the following morning in the same manner. This transient pain continued to return every morning, on assuming the erect position, until the summer of 1847, without the occurrence of any other symptom, such as swelling or tenderness, to indicate the nature or seat of the difficulty. This pain invariably returned on rising to my feet, after resting sometime in the horizontal position, but never continued to exceed one or two minutes. During this period, my heel gave me no uneasiness, my general health was good, with the exception of some dyspeptic symptoms for two or three years after the injury, which I attributed to sedentary habits, being then a student, and which passed off after engaging in practice.

In the summer of 1847, I began for the first time to experience a tenderness about the heel whenever it came in contact with solid substances with more than ordinary force, as in stamping or making a misstep. This tenderness gradually increased until I began to suffer some pain in walking at my usual pace, unless I was careful to slide my foot along without raising it but slightly from the ground. There was no appreciable swelling until December of that year, when, upon drawing on my boot one morning, I suffered so much pain that I was obliged to withdraw it immediately. During the day, the heel swelled considerably, and being hot and painful at night, I scarified it deeply and held it in warm water to favor the bleeding. On the following day, I was disappointed at finding my foot still worse, and was obliged to use a crutch in walking. Leeches and poultices were applied, and subsequently iodine and other medicinal applications were employed, but without even palliating the symptoms. Nothing but cold water and snow gave me even temporary relief; these were very grateful, and I resorted to them often, as the relief they afforded enabled me to attend to my business, though I suffered much pain night and day. I continued to grow gradually worse, until some time in February, 1848, when I determined to consult Dr. Parker. At this time there seemed to be a thickening of all the tissues about the os calcis, with a deep seated, constant pain, as if the fibrous tissue was put upon the stretch; occasionally the pain was lancinating.

Dr. Parker advised an incision down to the bone. which he accordingly made, dividing the periosteum of the os calcis about an inch and a half on its inferior surface; the wound bled freely and gave a little relief to the severity of the pain, but of short duration. I kept the wound discharging four or five weeks, but finding no material benefit, allowed it to heal. Soon after this, I applied moxas, by the advice of Dr. Ticknor, U. S. N., but with no benefit. The pain continued to increase in severity, being worse at night than during the day. I resorted to large doses of morphine to obtain sleep, and often two grains at a dose would produce only a short nap, from which I would be awakened by the most excruciating lancinating pains in the heel. I finally began the use of quinine in the summer of 1848, as I was beginning to emaciate and lose my appetite, taking eight grains per day, with cold applications to the affected part. Under this treatment my appetite and general health improved, and also the local difficulty; the pain was much less severe, and in a short time I threw aside my crutch, still continuing my practice, and got around very comfortably with a cane. This improvement lasted until December, 1848, when I had an attack of acute bronchitis, for which I was bled and took antimony, and of course omitted the quinine. I was confined to my bed about ten days, and in this time the pain in my heel became worse; on getting up I discovered for the first time, a tumor on the outer aspect of the heel, just below the external malleolus, which had a spongy, elastic, and pulsating feel, and was painful on pressure; the veins were much enlarged about the heel. I again resorted to the quinine, from which I had derived so much benefit, but it now gave me no relief.

I grew worse so rapidly, that I again visited New York with the intention of submitting to amputation, if this course was deemed advisable. I had come to the conclusion that I could not much longer survive such indescribable agony (I will not call it by so soft a name as pain). Dr. Parker advised immediate amputation, to which I cheerfully assented. He performed the operation on the 24th of January, 1849, after administering a mixture of chloroform and ether. I resumed the practice of my profession about three months after the operation, and have continued actively engaged since, in the enjoyment of good health. I have supplied my deficient extremity with an artificial limb, which answers its purpose admirably.

